# A comparison of the labeling of antineoplastic and supportive medicines in China, Japan, and the US based on the WHO essential medicines list

**DOI:** 10.3389/fmed.2025.1600207

**Published:** 2025-11-20

**Authors:** Chenyu Du, Jiaxin Liu, Caiyi Wang, Pengcheng Liu

**Affiliations:** School of International Pharmaceutical Business, China Pharmaceutical University, Nanjing, China

**Keywords:** drug labels, antineoplastic and supportive medicines, NMPA, PMDA, FDA

## Abstract

**Objective:**

This study systematically compares the safety information presented on drug labels for antineoplastic and supportive care medications listed on the WHO Essential Medicines List (EML) across three major pharmaceutical markets: China, Japan, and the United States.

**Methods:**

The safety information of the same drugs approved in the three countries was compared and analyzed, involving the calculation of the proportion of each safety information and the percentage of boxed warnings (PBW) to all information on the label, we also comparative analysis of pediatric medication information studied from each country.

**Results:**

There were substantial differences in each safety information of the labels in three countries. Except for the proportion of information on use in the elderly, the proportion of each type of safety information differed significantly between countries, and the content also varied widely. Concordance of the presence or the absence of a BW on the label between China, Japan, and the US was 36% on the total labels. Of the 19 drugs, Japan had the most pediatric drug information with 13 drugs, followed by the US with nine drugs and China with eight drugs.

**Conclusion:**

There were significant differences in the safety information on drug labels in China, Japan, and the US from the same manufacturer, which could pose a risk to patients. Regulatory agencies should better align their approaches to labeling to prevent confusion or misunderstanding across regions.

## 1 Introduction

In recent years, the number of cancer patients and the burden of cancer disease have increased worldwide (1). In 2019, the World Health Organization (WHO) released the latest WHO EML, adding 12 new cancer medicines to 48 antineoplastic and supportive medicines to help countries prioritize widely available and affordable cancer medicines. The global circulation and use of anticancer drugs have intensified, which has led to greater awareness of each other’s approaches, decision-making, and interactions seeking alignment among drug regulatory authorities around the world (2), which is essential as it allows for the assessment of drug safety and efficacy (3), as well as the oversight of drug labeling to provide health care professionals and patients with comprehensive information about the efficacy, safety, and use of drugs (4). Harmonized drug labels can ensure that everyone, regardless of region, receives the same comprehensive information necessary to make an informed decision (3).

However, there are differences in the content and regulation of labels from country to country, and researchers have tried to identify the causes of differences in drug labels between regions, with various results. One study found significant differences by comparing the safety information in labeling new molecular entities in the US, United Kingdom (UK), and Japan (5). Another study compared Food and Drug Administration (FDA) Package Inserts (PIs) and European Medicines Agency (EMA) Summary of Product Characteristics (SmPCs) and found poor consistency between the two regions (3). A comparison study of adverse drug reactions and boxed warnings on oncology drug labels in Japan and the US found significant differences in all sections of the labels between the two countries, due to differences in regulatory and historical factors in both local and global contexts (6). Some studies have found a low level of consistency of safety information in package inserts in Japan and the US and a low consistency between regulatory decisions on labeling changes and the timeliness of those changes (7, 8).

China consumes a lot of anti-cancer drugs every year, and the regulation and use of drug inserts deserve attention (9), but there are few studies on Chinese drug labels, so we chose the US and Japan, which are also member of the International Conference on Harmonisation of Technical Requirements for Registration of Pharmaceuticals for Human Use (ICH), to conduct a study on the comparison of anti-cancer drug labels. The three agencies conduct independent reviews of available data and have their guiding standards for labeling format and content; each regulatory agency’s approach, language, and recommendations for use may differ (10). Our objective was to compare Chinese, Japanese, and US labeling for the same drug to determine whether there are consistent similarities or differences and to provide an evidence base for better regulation of drug safety.

## 2 Methods

### 2.1 Data sources

This study was a cross-sectional study conducted in the period between June and October 2023, with data collection from June to August 2023. We searched which of the 60 antineoplastic and supportive drugs on the WHO EML were imported from China National Medical Products Administration (11) (NMPA). Then, according to the manufacturer of the drug imported in China, we went to the Pharmaceuticals and Medical Devices Agency (PMDA), and the DailyMed to find the drug label of the same manufacturer (CSL STYLE ERROR: reference with no printed form). If there is a drug from the same manufacturer that is not available in Japan or the US, we use the Japanese Orange Book or the FDA Orange Book to find the reference listed drug (RLD) labels to replace it (12, 13). The labels must be approved in NMPA and PMDA or the US FDA. Labels must be accessible in the public domain.

### 2.2 Variable definitions, evaluation, and analysis

We performed a direct comparison of the proportion of all information given to each safety information across the same drug and the proportion of the number of labels with box warnings or warnings to that of all labels in the three countries. For example, the proportion of adverse reaction information was calculated by dividing number of adverse reaction information words by the total number of safety information words. We defined the measures independently of language: for Chinese and English, we counted the number of words, and for Japanese, we counted the number of letters in the safety sections, and then divided this by the total number of words or letters on the label. Each number of safety information words was calculated based on the sections listed in Table 1 from each country.

**Table 1:** Drug label information for analysis.

Categories for analysis	Drug label sections		
	China	US	Japan
Serious warnings	Warnings	Warnings	1. Warnings
Safety information	Adverse reactions	6 Adverse reactions	11. Adverse reactions
	Contraindications	4 Contraindications	2. Contraindications
	Precautions	5 Warnings and Precautions	5. Precautions concerning indications 7. Precautions concerning dosage and administration 8. Important precautions 14. Precautions concerning use 15. Other precautions
	Pregnancy and lactation	8.1 Pregnancy 8.2 Lactation 8.3 Females and Males of Reproductive Potential	9.4 Persons with Reproductive Potential 9.5 Pregnant Women 9.6 Brest-feeding Women
	Pediatric Use	8.4 Pediatric Use	9.7 Pediatric Use
	Geriatric Use	8.5 Geriatric Use	9.8 Geriatric Use
	Drug Interaction	7 Drug Interaction	10. Interactions
	Overdose	10 Overdose	13. Overdose
	Pharmacology and toxicology	12.1 Mechanism of Action 12.2 Pharmacodynamics 13 Nonclinical toxicology	18. Pharmacology

Pediatric use is often omitted in oncology labels. We narrowed the analysis to drugs with explicit pediatric information (dosage/age) in ≥1 country. We conducted a comparative analysis of pediatric medication information for 19 medicines across three countries, including information such as pediatric medication dosage and age on the label. If there is information on this drug for children, we mark it as 1, and if the trial was not conducted or the safety is unclear, we mark it as 0. Finally, we added up the scores of all 19 medicines in each country, and the higher the score, the more complete pediatric medication information in that country (Table 2).

**Table 2:** Comparative analysis of pediatric labeling information.

Drug	Country	Pediatric medication information	Pediatric age	Pediatric dose
Bleomycin	China	0		
	US	0		
	Japan	1		Conversion by body surface area and age
Carboplatin	China	0		
	US	0		
	Japan	1		Conversion by body surface area and weight
Cyclophosphamide	China	1		Conversion by body surface area
	US	1		Conversion by weight
	Japan	1		Conversion by body surface area
Cytarabine	China	1		Conversion by body surface area
	US	1		Conversion by weight
	Japan	1		Conversion by weight
Etoposide	China	0		
	US	0		
	Japan	0		
Ifosfamide	China	1		Conversion by body surface area
	US	0		
	Japan	1		Increase or decrease according to age and symptoms
Irinotecan	China	0		
	US	0		
	Japan	0		
Methotrexate	China	1	0 to 18 years old	Conversion by age
	US	1	0 to 18 years old	Conversion by age
	Japan	1		Conversion by age
Paclitaxel	China	0		
	US	0		
	Japan	0		
Procarbazine	China	0		
	US	0		
	Japan	0		
Dashatini	China	0		
	US	1	≥ 1 year old	
	Japan	1		Conversion by weight
Everolimus	China	1	≥ 1 year old	
	US	1	≥ 1 year old	
	Japan	1	≥ 1 year old	
Imatinib	China	1	≥ 3 years old	Conversion by body surface area
	US	1		Conversion by body surface area
	Japan	1		Conversion by body surface area
Nilotinib	China	1	≥ 2 years old	Conversion by body surface area
	US	1	≥ 2 years old	Conversion by body surface area
	Japan	1	≥ 1 year old	Conversion by body surface area
Rituximab	China	0		
	US	1	≥ 2 years old	
	Japan	0		
Dexamethasone	China	0		
	US	0		
	Japan	0		
Methylprednisolone	China	1		Based on age, physical fitness
	US	1	≥ 2 years old	Reduction of dosage
	Japan	1		Reduction of dosage
Mesna	China	0		
	US	0		
	Japan	0		
Rasburicase	China	1		Conversion by weight
	US	1		Conversion by weight
	Japan	1		Conversion by weight
Total score	China	9		
	US	10		
	Japan	12		

Warnings convey important safety information in eye-catching fonts on the first page of labels, alerting healthcare professionals and patients to medication risks. We identified a boxed warning (BW) on Chinese, Japanese, and US drug labels through a manual search and measured PBW on all labels.

### 2.3 Comparison of drug label safety information

There is a possibility that the volume of text is different, but the information conveyed is the same (14). To understand the differences in the information provided across the three countries, we read the content of each safety information, gave the complete content a score of 1, the incomplete or missing content a score of 0, and then summed the scores of each item; the higher the score, the more complete the content of each safety information (Table 3).

**Table 3:** Each safety information total score in China, Japan, and the US.

Item	Adverse reactions	Contraindications	Precautions	Pregnancy and lactation	Pediatric use	Geriatric use	Drug interaction	Overdose	Pharmacology and toxicology	Total
China	22	18	15	21	12	22	21	31	31	193
Japan	19	28	28	19	16	18	26	5	12	171
US	24	8	12	32	25	25	7	23	24	179

### 2.4 Statistical analysis

Descriptive statistics were performed on the data obtained. Data were presented as the mean and standard deviation (SD). One-way analysis of variance (One-way ANOVA) was performed to find out the differences between groups followed by Scheffe’s *post hoc* test to determine which means were different with a level of significance set at *p* < 0.05. Data were analyzed using Statistical Analysis Software (SPSS 26.0).

## 3 Results

Of the 60 drugs, 35 drugs were imported into China, but 2 drugs were excluded because no labels were found for the drugs in China. According to the manufacturer of 33 drugs, we went to PMDA and DailyMed to find the drug label of the same manufacturer and found 15 drug labels. For an additional 18 drugs, we used the reference-listed drug labels to replace them. Finally, 33 drug groups were finally included in our study.

The total number of label words on the Chinese labels {16,003 (10178) [mean (standard deviation)]} was higher than the number of Japan [12,196 (6406)] and the US [12,278 (8998)] label words with a *p*-value> 0.05. The total number of safety words was higher on Chinese labels [8,903 (5019)] when compared to Japan [4,829 (2616)] and the US [5,304 (4194)] label safety words with a p-value < 0.05.

### 3.1 Proportion of each safety information

Table 4 shows the proportion of each safety information on drug labels in three countries. The proportion of each safety information was significantly different across the countries except for the proportion of geriatric use information.

**Table 4:** Proportion of each safety information on the label.

Item	Adverse reactions	Contraindications	Precautions	Pregnancy and lactation	Pediatric use	Geriatric use	Drug interaction	Overdose	Pharmacology and toxicology	Total
China	23.1 [10.2]^#^	0.9 [0.7]	17.1 [0.9]^@^	3.0 [2.0]	0.8 [0.8]	0.9 [0.7]	5.0 [4.8]^@^	1.9 [1.9]^^^	6.0 [4.0]^#^	58.6 [12.6]^^^
Japan	13.9 [6.9]	1.4 [1.4]^@^	13.3 [5.5]	2.5 [1.5]	1.0 [1.7]	0.8 [0.6]	5.2 [4.8]^@^	0.2 [0.9]	3.8 [2.6]	40.6 [12.9]
US	19.0 [8.0]^#^	0.6 [0.8]	10.8 [7.5]	3.9 [2.1]^#^	2.0 [2.5]*	1.4 [1.6]	1.5 [1.8]	0.9 [1.0]^#^	4.4 [3.0]	43.7 [10.4]
*p* value	0.00	0.01	0.00	0.01	0.02	0.09	0.00	0.00	0.03	0.00

#Lager than Japan. @Larger than US. *Larger than China. ^Lager than Japan and US.

**Figure 1:**
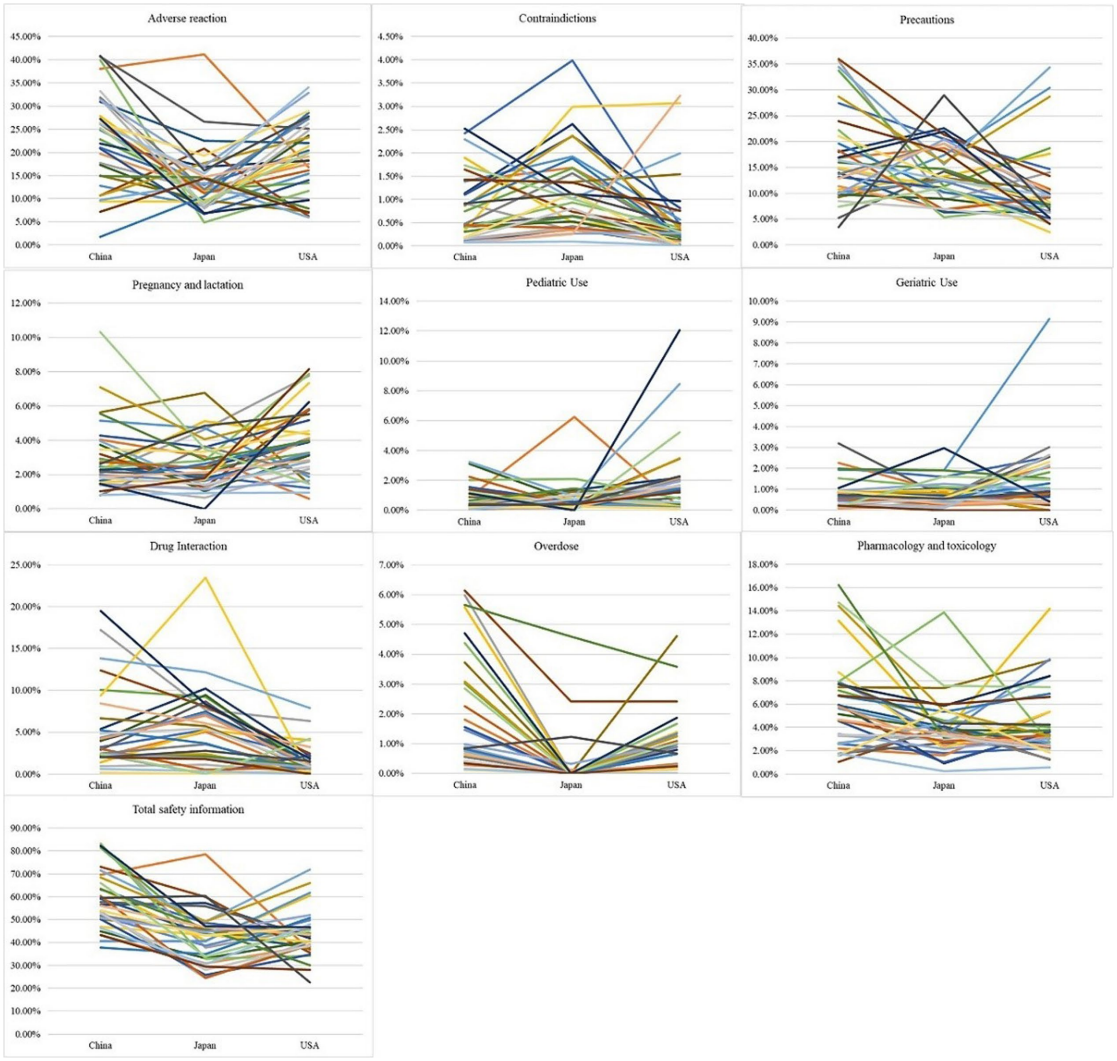
The proportion of each safety information on the label.

The proportion of adverse reaction information on Chinese labels {23.1 (10.2) [mean (standard deviation)]} and US labels (19.0 [8.0]) was higher than on Japanese labels [13.9 (6.9)]. The proportion of contraindication information on Japanese labels [1.4 (1.4)] was higher than on US labels [0.6 (0.8)]. The proportion of precaution information on Chinese labels [17.1 (0.9)] was higher than on US labels [10.8 (7.5)]. The proportion of pregnancy and lactation information on US labels [3.9 (2.1)] was higher than on Japanese labels [2.5 (1.5)]. The proportion of pediatric use information on US labels [2.0 (2.5)] was higher than on Chinese labels [0.8 (0.8)]. The proportion of drug interaction information on Chinese labels [5.0 (4.8)] and Japanese labels [5.2 (4.8)] was higher than on US labels [1.5 (1.8)]. The proportion of overdose information on Chinese labels [1.9 (1.9)] was higher than on Japanese labels [0.2 (0.9)] and US labels [0.9 (1.0)]. The proportion of pharmacology and toxicology information on Chinese labels [6.0 (4.0)] was higher than on Japanese labels [3.8 (2.6)]. The proportion of total safety information on Chinese labels [58.6 (12.6)] was higher than on Japanese labels [40.6 (12.9)] and US labels [43.7 (10.4)] (Figure 1).

### 3.2 Comparison of drug label safety information

Table 5 shows a detailed comparative analysis of the content of each safety information. We sum the scores of each safety information and then get the total score for each item in three countries (Table 3). Chinese labels achieved the highest scores in overdose, pharmacology, and toxicology, suggesting that these sections are more comprehensively labeled. Japan scored highest in contraindications, precautions, and drug interactions, while the US received the highest scores in adverse reactions, pregnancy and lactation, pediatric use, and geriatric use. Overall, Chinese drug label safety information total scores are higher than those in the US and Japan.

**Table 5:** Comparative analysis of the content of each safety information in China, Japan and the USA.

Drug	Country	Adverse reactions	Contraindications	Precautions	Pregnancy and lactation	Pediatric use	Geriatric use	Drug interaction	Overdose	Pharmacology and toxicology
Bleomycin	China	1	1	1	1	0	0	0	1	1
	USA	0	0	0	1	0	1	1	0*	1
	Japan	1	1	1	1	1	0	0	0*	1
Carboplatin	China	1	1	0	1	0	1	1	1	1
	USA	0	0	0	0	0	1	0	0	0*
	Japan	1	1	1	1	1	1	0	0	0*
Cyclophosphamide	China	0	0	0	0	0	0	0	1	1
	USA	1	0	0	1	1	1	0	1	0
	Japan	1	1	1	0	1	1	1	0*	0*
Cytarabine	China	0	1	0	1	1	1	0	1	1
	USA	1	0	0	1	0	0*	0	1	0
	Japan	1	0	1	1	0	1	1	0*	0*
Etoposide	China	0	0	0	1	1	0	0	1	1
	USA	1	0	0	1	1	1	0	1	0*
	Japan	1	1	1	1	1	0	1	0*	0*
Ifosfamide	China	1	1	0	0	0	0	0	1	1
	USA	1	0	0	1	0	1	0	0	1
	Japan	0	0	1	0	1	0	1	0*	0*
Irinotecan	China	0	1	0	1	0	1	0	1	1
	USA	1	0	0	1	0	1	0	1	1
	Japan	1	1	1	1	1	0	1	0	0*
Methotrexate	China	0	1	0	1	0	1	1	1	1
	USA	1	0	0	1	1	0	0	0	1
	Japan	1	0	1	1	0	1	1	1	0*
Paclitaxel	China	0	0	0	1	1	1	0	1	1
	USA	0	0	0	1	1	1	0	1	1
	Japan	1	1	1	0	0	0	1	0*	0*
Procarbazine	China	1	1	0	1	0	1	1	1	1
	USA	1	0	0	1	1	0	0	1	1
	Japan	0	0	1	1	0	0	0	0*	0*
Dashatini	China	1	0	1	0	0	1	1	1	1
	USA	1	0	1	1	1	1	1	1	0
	Japan	0	1	0	0	0	1	1	0*	0
Everolimus	China	1	1	1	1	1	1	1	1	1
	USA	1	0	1	1	1	1	0	0*	1
	Japan	0	1	1	0	0	0	1	0*	1
Imatinib	China	1	0	1	1	1	1	1	1	1
	USA	1	0	1	1	1	1	0	1	1
	Japan	0	1	1	1	1	1	1	0*	0
Nilotinib	China	1	1	1	1	0	1	1	1	1
	USA	1	1	1	1	1	1	1	1	0
	Japan	1	1	0	0	0	1	1	1	0
Rituximab	China	1	0	1	0	0	0	1	1	1
	USA	1	0	1	1	1	1	0	0*	1
	Japan	0	1	1	0	1	0	1	0*	1
Dexamethasone	China	0	0	1	0	1	1	1	1	1
	USA	1	1	0	1	0	0	0*	0*	0
	Japan	1	1	1	0	1	1	1	0*	0
Methylprednisolone	China	0	1	1	1	1	1	1	1	0
	USA	0	1	0	1	1	1	0	0	1
	Japan	1	1	0	1	1	0	1	0*	1
mesna	China	1	1	1	0	1	1	1	1	1
	USA	0	0	1	1	0	1	0	1	1
	Japan	0	1	0	0	1	0	1	0*	1
Rasburicase	China	1	0	1	1	1	1	1	1	1
	USA	1	0	0	1	1	0	1	1	1
	Japan	0	1	0	1	0	0	0*	0*	1
Bendamustine	China	0	0	0	0	1	1	0	1	1
	USA	0	0	0	1	1	1	0	1	0
	Japan	1	1	1	1	0	1	1	0*	0*
Docetaxel	China	1	1	0	0	0	0	0	1	1
	USA	0	0	0	1	1	1	0	1	1
	Japan	0	1	1	1	0	0	1	0*	0*
Fludarabine	China	0	0	0	1	0	1	1	1	1
	USA	0	0	0	1	1	0	0	1	0
	Japan	1	1	1	1	0	1	1	0*	0*
Gemcitabine	China	1	0	0	0	0	1	0	1	1
	USA	1	0	0	1	1	1	0*	1	1
	Japan	0	1	1	0	0	1	1	0	0*
Melphalan	China	0	0	0	1	0	1	1	1	1
	USA	0	0	0	1	0	1	0	1	1
	Japan	1	1	1	1	1	1	1	1	0*
Bortezomib	China	1	0	0	1	0	1	1	1	1
	USA	1	1	0	1	1	1	0	1	1
	Japan	0	0	1	1	0	1	0	1	0*
Erlotinib	China	1	1	0	1	0	1	0	1	1
	USA	0	0	1	1	1	1	0	1	1
	Japan	1	1	1	1	0	1	1	0*	0*
Ibrutinib	China	1	0	1	1	0	1	1	1	1
	USA	1	0*	1	1	1	1	0	1	1
	Japan	0	1	1	0	1	1	1	0*	1
Trastuzumab	China	1	0	1	1	1	1	1	0	1
	USA	1	0*	1	1	1	1	1	1	1
	Japan	1	1	1	1	1	1	0*	0*	1
Nivolumab	China	1	1	1	1	0	0	1	1	1
	USA	1	0	1	1	1	1	0*	0*	1
	Japan	1	1	1	1	1	0	1	0*	0
Anastrozole	China	1	1	1	1	0	0	0	1	1
	USA	1	1	1	1	1	1	1	1	1
	Japan	0	1	1	1	0*	1	0*	0*	1
Bicalutamide	China	1	1	0	0	0	0	1	1	0
	USA	1	1	0	1	1	0	0	1	1
	Japan	1	1	1	0*	0*	1	1	0*	1
Leuprorelin	China	1	1	1	0	1	0	1	0	1
	USA	1	1	0	1	1	0*	0*	0	1
	Japan	1	1	1	0	1	0*	1	0*	1
Zoledronic acid	China	1	1	0	0	0	0	1	1	1
	USA	1	1	1	1	1	1	1	1	1
	Japan	0	1	1	0	0	0	1	1	1

1 The item is complete. 0 The item is incomplete. 0* The item is missing.

### 3.3 Labels with a warning

Table 6 shows the proportion of the number of labels with BW in China, Japan and the US. PBW on the Japanese labels (85%) was larger than that on the US labels (55%) and Chinese labels (21%). Concordance of the presence or the absence of a BW on the label between China, Japan, and the US was 36% on the total labels.

**Table 6:** Proportion of the number of labels with a boxed warning in China, Japan and the US.

Country	Number (n = 33)
China	21 (7)
Japan	85 (28)
US	55 (18)
China^+^/Japan^+^/US^+^	21 (7)
China^—^/Japan^+^/US^+^	33 (11)
China^—^/Japan^+^/US^—^	45 (15)
China^+^/Japan^+^/US Concordance*	36 (12)

Values are % (number). +Represents labels with a boxed warning. —Represents labels without a boxed warning. *Concordance represents the sum of labels with a boxed warning both in the three countries and those without a boxed warning in either of the three countries.

### 3.4 Comparative analysis of pediatric medication information

We compared and analyzed the differences in pediatric medication information for 19 medicines included in the WHO EMLc in three countries (Table 2). Of the 19 drugs, Japan had the most pediatric drug information with 13 drugs, followed by the US with nine drugs and China with eight drugs. And seven out of the 19 drugs have pediatric medication information available in all three countries.

## 4 Discussion

### 4.1 Proportion of each safety information

We found significant differences in the proportion of each safety information provided on the same drug label in the three countries, except for geriatric use information. Chinese labels have significantly more proportion in more safety information items than other countries, followed by the US and then Japan, and Chinese labels’ proportion of total safety information is significantly more than the US and Japan. In a previous study conducted on the labels of new molecular entities between the US, the UK, and Canada, it was found that significant differences among the mean proportion of total safety information (PSI) of all three countries (14).

In our study, we also found that there are significant differences among the mean PSI and proportion of many safety information items of all three countries, which is most likely due to the different guidelines set by these authorities for the preparation of drug labels (15–17), as different laws in different countries may influence regulatory decisions (18).

### 4.2 Comparison of drug label safety information

The proportion of each safety information was generated by counting words/characters, which are crude measurements that do not always indicate critical safety issues (5). So we compared the content of each label’s safety information and found that there were indeed differences between labels in three countries. We found that except for precautions, Chinese labels have significantly more words than Japanese labels, but the score is lower than that of Japan, that is, the information integrity is lower than that of Japan, and the other safety information is higher the number of words, the higher the score.

The Japanese guidelines for writing package labels require precautions to be divided into [precautions related to indications], [precautions related to use and dosage], [important basic precautions], [other precautions], [operational precautions], etc., while the Chinese guidelines for writing package labels do not require precautions to be divided, which may lead to a more complete content of precautions in Japanese package leaflets (15, 16).

We went on to study the section related to adverse reactions in China, Japan, and the US. The proportion of adverse reaction information on Chinese labels and US labels was higher than on Japanese labels and the adverse reaction score on Chinese labels and the US labels was also higher than those in Japanese labels. For the drug mesna, the proportion of adverse reaction information on Chinese labels (0.37) and US labels (0.12) was higher than on Japanese labels (0.05), and the Chinese label contained 11 adverse reactions, the US 15, and Japan only 10.

In the pregnancy and lactation, pediatric use, and geriatric use sections, we also found that the US, which had more words, scored higher. We found that the Chinese and Japanese labels contained vague information about the drug details for certain groups, including that the trial was not conducted, that there were no references, that it was not clear, and so on. For example, there is no data on the presence of everolimus or its metabolites in human milk, the effects of everolimus on the breastfed infant, or on milk production. The lack of human data relevant to pregnant and lactating populations, children and the elderly has long been identified as an area of significant public health need (19), mainly because the current patients participating in pre-marketing clinical trials of drugs are highly selected patients, only qualified patients can participate in clinical trials, and there will be a lack of clinical data for special populations (20).

### 4.3 Labels with a warning

The warning is displayed in a prominent font below the title of the drug label, which is about serious adverse reactions to medicines and their serious safety concerns, covering items such as contraindications and precautions. Our comparison shows that Japan has a higher proportion of BWs, followed by the US and China. In addition, the concordance of the presence or the absence of a BW on the label between China, Japan, and the US was 36% on the total labels, indicating a low level of coordination between the three countries, which could have implications for medical professionals and patients (3).

Both the US and Japan have stricter requirements for warnings, and contraindications or serious warnings, especially those that could lead to death or serious injury, must be included in boxed warnings. China’s Provisions for Drug Insert Sheets and Labels stipulate that drug manufacturers can take the initiative to add warnings to drug specifications or labels, but the regulations are not sufficiently clear on the legal responsibility for regulating warnings and do not stipulate that regulatory authorities, production and management companies have their responsibilities and must do so. The previous study found that the use of febuxostat decreased significantly in the US after the FDA added a boxed warning to the label for febuxostat (21). Physicians may have responded to the FDA’s warning by prescribing AAPs less often and adopting, instead, alternative therapeutic strategies for managing NPSs in elderly individuals with dementia (22). Therefore, many Chinese pharmaceutical companies do not actively add warnings to their drug labels so as not to affect drug sales.

### 4.4 Comparative analysis of pediatric medication information

We found that only 7 of the 19 WHO EMLc drugs in three countries had pediatric medication information on their use in children, confirming previous research that pediatric drug use is often missing due to lack of evidence or regulatory delays in label updates, which may put children at higher risk for side effects, adverse drug reactions, and medication errors (23).

We also found differences in pediatric dosing regimens of the same drug in different countries, for example, cytarabine for children with acute leukemia, China uses body surface area to convert drug doses, while the United States and Japan use body weight to convert drug doses. Historically, however, pediatric dosing regimens have often been derived from pharmacokinetic data in adults (24). This approach, although clinically widespread, is not supported by solid empirical evidence and may result in neonates and children being exposed to inappropriate systemic drug levels (25).

While pediatric drug development is international in scope, pediatric drug labeling information is not uniformly shared and is under the control of national regulatory authorities (4), requiring countries to increase cooperation, information sharing, dissemination, discussion, and efforts to reach consensus on regulatory “standards” for quality pediatric medicines.

### 4.5 Limitations

We acknowledge several limitations of our study. The most important limitation was the generalizability of our findings in antineoplastic and supportive medications to other types of medications. Also, this study included few drug samples, which may have affected the accuracy of the results.

## 5 Conclusion

There were significant differences in the safety information on drug labels in China, Japan, and the US from the same manufacturer, which could pose a risk to patients. The amount and content of information on drug labels vary from country to country. Regulatory agencies should better align their approaches to labeling to prevent confusion or misunderstanding across regions. This could be done in a variety of forums, including bilaterally as issues arise, starting with an understanding of the thinking underlying our differences, but eventually through standard-setting organizations.
